# Analysis of tRNA halves (tsRNAs) in serum from cattle challenged with bovine viral diarrhea virus^#^

**DOI:** 10.1590/1678-4685-GMB-2018-0019

**Published:** 2019-06-27

**Authors:** Tasia M. Taxis, Fernando V. Bauermann, Julia F. Ridpath, Eduardo Casas

**Affiliations:** 1 National Animal Disease Center, USDA, ARS, Ames, IA, USA

**Keywords:** Cattle, Bovine Viral Diarrhea Virus, transfer RNA, transfer RNA halves, small non-coding RNA

## Abstract

Acute infections of bovine viral diarrhea virus (BVDV) lead to a range of clinical presentations. Laboratory tests for detection depend on collection of samples during a short viremia. Acutely infected animals remain largely undiagnosed. Transfer RNA halves (tsRNAs) are hypothesized to function like microRNAs to regulate gene expression during an immune response. The objective of this study was to identify tsRNAs in cattle that had been challenged with a non-cytopathic field strain of BVDV. Colostrum-deprived neonatal Holstein calves were either challenged with BVDV (n=5) or mock challenged (n=4). Sera was collected prior to challenge and days 4, 9, and 16 post challenge. RNA was extracted and read counts of small non-coding RNAs were assessed using next-generation sequencing. A total of 87,838,207 reads identified 41 different tsRNAs. Two 5’ tsRNAs, tsRNA^ProAGG^ and tsRNA^ValAAC^, differed across time. Two 5’ tsRNAs, tsRNA^GlyCCC^ and tsRNA^GlyGCC^, differed between treatment groups across time. Four days post challenge, 5’ tsRNA^GlyCCC^ and tsRNA^GlyGCC^ were significantly lower in the challenged group than the control group. Further studies are needed to identify the importance and function of 5’ tsRNA^GlyCCC^ and tsRNA^GlyGCC^ in serum samples of cattle challenged with BVDV.

## Introduction

Bovine viral diarrhea viruses (BVDV) consists of two species within the Pestivirus genus. Infection can lead to respiratory, enteric, and reproductive disease in cattle accompanied by immune suppression and increased susceptibility to subsequent viral or bacterial infections. Signs of an infected animal range from nasal discharge, cough, pyrexia, and depression to diarrhea, hemorrhagic syndrome and death (Houe, 2003; Ridpath and Fulton, 2009). In many cases, signs of BVDV infection are subclinical, and are not recognized as a result of BVDV infection. Therefore, resulting in an underestimation of BVDV infections (Houe, 2003; Hessman *et al.*, 2009; Ridpath and Fulton, 2009; Walz *et al.*, 2010). Acute, or transient infections commonly last from 10 to 14 days before the virus is cleared (Hessman *et al.*, 2009). Clinical signs, when present, are typically observed between 3 and 9 days post infection (Hessman *et al.*, 2009). However, the animal remains immunosuppressed following infection, making a BVDV infected animal more susceptible to subsequent infections with other pathogens (Ridpath, 2010). It is theorized that BVDV infections contribute to bovine respiratory disease complex (BRDC) by potentiating and increasing the severity of secondary infections (Ridpath, 2010). Identifying animals that have had a BVDV infection, or a suppressed immune system, and are at risk to develop respiratory disease could improve herd health management and control BRDC.

Small non-coding RNAs, such as tRNA halves (tsRNAs) or tRNA-derived fragments (tRFs), are present in serum and have been associated with gene regulation and disease (Lee *et al.*, 2009; Casas *et al.*, 2015; Venkatesh *et al.*, 2016). The cleaving of a mature tRNA molecule into fragments of 28 to 36 base pairs in length leads to two fragment types; a 3’ and a 5’ tRNA half (tsRNA) (Garcia-Silva *et al.*, 2012; Deusch *et al.*, 2015; Kanai, 2015; Diebel *et al.*, 2016). While the particular function of tsRNAs are unknown, studies support the hypothesis that tsRNAs are similar to microRNAs in regulating gene silencing and cell proliferation (Haussecker *et al.*, 2010; Garcia-Silva *et al.*, 2012; Dhahbi, 2015). In particular, extracellular tsRNAs, such as those found in serum, have been associated with stress, immunity, tumor growth, and viral infections (Thompson *et al.*, 2008; Dhahbi *et al.*, 2013; Wang *et al.*, 2013; Green *et al.*, 2016; Li *et al.*, 2018). Most of the available research has been conducted *in vitro* using cultured cells, while *in vivo* investigation, using an animal model, is scarce (Cole *et al.*, 2009; Dhahbi *et al.*, 2013; Wang *et al.*, 2013; Diebel *et al.*, 2016). Therefore, the objective of this study was to identify tsRNAs in serum of calves acutely infected with a non-cytopathic field strain of BVDV (BVDV2-RS886).

## Material and Methods

### Animals

In order to avoid interference from virus-specific humoral or cellular immunity, all animals were colostrum-deprived male Holstein calves between 3 and 5 weeks of age at the start of the study. Calves were sorted into either a mock inoculated (control group) or BVDV challenged group (challenged group). This study was performed as two replicates. In both replicates, the control group (n=4) was mock inoculated on day 0 (non-infected MDBK cells), and the challenged group (n=5) was exposed to a non-cytopathic field strain of BVDV (BVDV2-RS886) on day 0. In the first replicate there were 2 mock- and 3 BVDV-challenged animals. In the second replicate there were 2 mock- and 2 BVDV-challenged animals. Viral inoculations consisted of 4 mL of viral preparation (1 X 10^6^ TCID/mL), and mock inoculations consisted of clarified freeze/thaw lysate of MDBK cells. All inoculations were delivered by direct instillation of the inoculation in the nose. Calves were observed twice daily for signs of respiratory disease. Blood was collected 1 to 2 days prior to the initial inoculation (here on referred to as day 0) and days 4, 9, and 16 post inoculation.

Blood samples were collected via jugular venipuncture in SST vacutainer tubes (BD, Franklin Lakes, NJ, USA). The tubes were then incubated at 37°C for 30 min., centrifuged at 1250 x g for 30 min., and aliquoted serum samples were stored at -80°C until processed.

Housing, care, and sample collection from animals was performed according to the management protocol approved by the Animal Care and Use Committee of the National Animal Disease Center in Ames, Iowa, USA (ARS-2667).

### Virus characterization and propagation

The BVDV2-RS886 strain was first isolated from a persistently infected calf (Liebler-Tenorio *et al.*, 2004). It is a non-cytopathic strain that belongs to the BVDV2 species. Under controlled conditions, experimental infection of colostrum deprived calves with BVDV2-RS886 results in low-grade pyrexia and reduced circulating lymphocytes (Ridpath *et al.*, 2013). For the present study, virus amplification and titration was performed using Madin Darby bovine kidney (MDBK) cells. Cells were free of ruminant pestivirus antigens and antibodies. Detailed procedures for virus amplification are described by Ridpath *et al.* (2013).

### Small non-coding RNA isolation, library preparation, and sequencing

Total RNA was purified from serum samples using the miRNeasy Serum/Plasma Kit (QIAGEN, Germantown, MD, USA) and were eluted in 14 μL RNase-free water. The concentration of small RNAs that were extracted in each sample was determined using a 10-40 nucleotide gate on an Agilent 2100 Bioanalyzer Small RNA chip (Agilent Technologies, Santa Clara, CA, USA).

Six microliters (6 μL) of small RNA from each extraction was used to prepare individual libraries using the NEBNext Multiplex Small RNA Library Prep Kit (New England BioLabs, Ipswich, MA, USA) and Illumina 1-23 indexed primers. Library purification was performed using the QIAquick PCR purification kit (QIAGEN). Each library was run on an Agilent 2100 Bioanalyzer High Sensitivity DNA chip (Agilent Technologies) to determine quality and quantity of RNA between 135-170 base pairs. Then, 30 ng of each library was pooled. Two library pools were created. One library pool contained 17 libraries from this study, and the second library pool contained 19 libraries from this study. Both library pools contained 23 libraries each, with additional libraries added to the pool from similar concurrent experiments. For example, all libraries were created from viral challenged/control cattle serum samples following the described methods in the current manuscript. Each library pool was size selected (142-170 nt) using the Pippin Prep (SAGE Sciences, Beverly, MA, USA) on a 3% agarose gel without added ethidium bromide. Following size selection, library pools were concentrated using the QIAquick PCR purification kit (QIAGEN) and eluted in RNase-free water. An Agilent 2100 Bioanalyzer High Sensitivity DNA chip (Agilent Technologies) was used to determine the concentration of each library pool between 135-170 base pairs. Library pools were stored at -20 °C until sequencing.

Size selected library pools were sequenced as single-end 50 base pair reads using the Illumina HiSeq 2500 System (Illumina, San Diego, CA, USA) in the Sequencing Core Facility at Iowa State University (Ames, Iowa). Nucleotide sequence data is available in the NCBI SRA database under the BioProject accession number SRP091488.

### Data and statistical analysis

FastQC v0.11.2 **(**http://www.bioinformatics.babraham.ac.uk/projects/fastqc) and fastx_clipper program in a fastx toolkit (http://hannonlab.cshl.edu/fastxtoolkit/) was used to determine the quality of the Illumina reads and remove the adapter sequence from each read, respectively. Unique reads were collapsed using a custom script, and reads 18 to 50 base pairs in length were retained for analysis (Baras *et al.*, 2015). These reads were mapped to the *Bos taurus* genome (ENSEMBL UMD3.1.75) using Novoalign software (Novocraft Technologies, Petaling Jaya Selangor, Malaysia), allowing two mismatches. *Bos taurus* genome aligned reads were then aligned to a database containing different annotated genome features in order to determine the aligned reads’ origin: genomic tRNA sequences were downloaded from the UCSC genomic tRNA database (http://gtrnadb.ucsc.edu/). Mitochondrial tRNA, cDNA, and other non-coding RNA sequences were downloaded from ENSEMBL version 75. The Illumnia reads that aligned to tRNA genes, or their flanking sequences were further characterized. First, these reads were aligned to a *Bos taurus* tRNA database using BLASTN and the results were processed using a custom script. Reads that aligned perfectly to the 5’ end of a mature tRNA were classified as 5’ tsRNAs, and reads that aligned perfectly to the 3’ end of a mature tRNA were classified as 3’ tsRNAs. After reads of 5’ tsRNAs and 3’ tsRNAs were determined, the number of reads per serum sample was obtained using a custom script, and normalization of library size to reads per million (RPM) was obtained for statistical analysis. Statistical analysis included tsRNAs with ≥ 5,000 total reads (Table S1).

Data was analyzed as a mixed model using SAS (SAS Inst. Inc., Cary, NC). The main effects analyzed were time (day 0, 4, 9, and 16), treatment (challenged or control), and the interaction between treatment and time. Read counts on day 0 were included as a covariate. Random effects included the replicate of the study (1 or 2), and animal ID was run as a repeated measure. Uncorrected significances are presented as the study was designed to ascertain nominal significant differences with the minimal number of animals. Significant values should be taken into consideration when interpreting results.

## Results

All challenged animals developed pyrexia and leukopenia starting at day 3 post challenge. Pyrexia resolved by day 7, and leukopenia was resolved by day 9. Control animals showed no signs of pyrexia or leukopenia and were considered healthy.

A total of 191,073,075 reads were obtained from sequencing of which 14,348 reads aligned to 3’ tsRNAs and 87,838,207 reads aligned to 5’ tsRNAs. Additional small non-coding RNAs (sncRNAs) were also identified including microRNAs (905,861 reads), small nuclear RNAs (208,440 reads), small nucleolar RNAs (143,879 reads), mitochondrial tRNAs (17,718 reads), and cDNAs (410,395 reads).

Forty-four different anticodons of 3’ tsRNAs were identified, of which none had greater than 5,000 total reads. Read lengths ranged from 28 to 50 base pairs. Four 3’ tsRNAs with read counts ≥ 1,000 (ranging from 1,246 to 2,956) were analyzed. None of them were significantly different across time, between treatments, or between treatments across time.

Forty-one different anticodons of 5’ tsRNAs were identified, of which 18 had ≥ 5,000 total reads across all samples. Four 5’ tsRNAs, tsRNA^ProAGG^, tsRNA^ValAAC^, tsRNA^GlyCCC^, and tsRNA^GlyGCC^ were significantly different either across time or between treatments across time, as described below (Table S2). None of the analyzed 5’ tsRNAs were significantly different between treatment groups, regardless of time.

5’ tsRNA^ProAGG^ and tsRNA^ValAAC^ significantly differed across time (Table 1). For 5’ tsRNA^ProAGG^ (*p*=0.03) the count number increased from day 0 to day 9 and then decreased on day 16. For 5’ tsRNA^ValAAC^ (*p*=0.03) a repeated rise and lowering pattern was observed. The count number doubled from day 0 to day 4, then halved to match day 0 on day 9, and increased to resemble day 4 levels on day 16.

**Table 1:** 5’tRNA-derived RNA halves, normalized mean for time of serum collection, standard error (SE), and their association (p-value) across all animals.

	Time of serum collection (RPM)^*^					
5’ tRNA half	Day 0^1^	Day 4	Day 9	Day 16	SE	*p*-value
tsRNA^ProAGG^	393^a, b^	600^a.c^	645^b^	641^c^	94	0.03
tsRNA^ValAAC^	37^a^	74^a, b^	42^a^	94^b^	15	0.03

* ^*^ RPM stands for reads per million

1 Day 0 samples were collected 1-2 days prior to challenge

a, b, c, Means (RPM) without a common superscript within row are statistically different (*p* ≤ 0.05).

**Figure 1:**
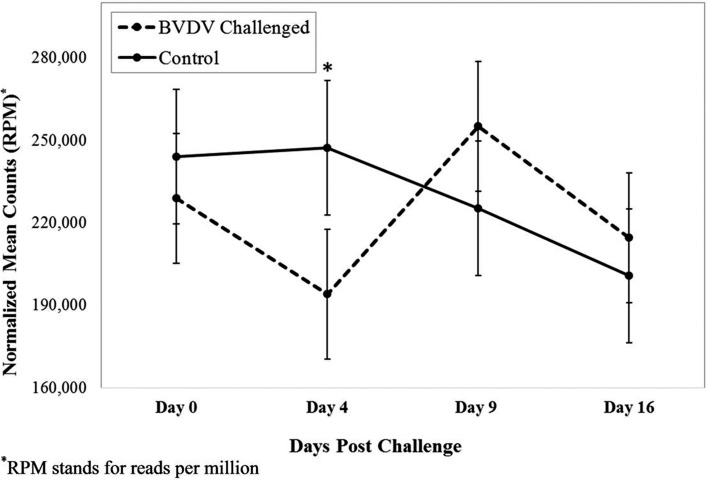
Interaction of time by treatment for 5’ tsRNA^GlyCCC^ (*p* = 0.03). Dashed line corresponds to cattle challenged with bovine viral diarrhea virus (BVDV). Solid line corresponds to control animals. Error bars represent standard error bars of the normalized means. An asterisk above a time point represents a significant (*p* = 0.01) difference in number of read counts between BVDV challenged and control animals at that time point.

Figure 1 depicts the profile for 5’ tsRNA^GlyCCC^ by treatment group across time. A significant difference in count number between the challenged and control groups was observed 4 days after initial inoculation. The control group had a greater count number (*p*=0.01) when compared to the challenged group. While a significant difference between the two treatment groups was similar at other times, the count number in the control group was higher than the challenged group from day 0 to day 4. Between day 4 and 9, the count number increased in the challenged group and decreased in the control group, resulting in the challenged and control groups to have a similar count number by day 9.

**Figure 2:**
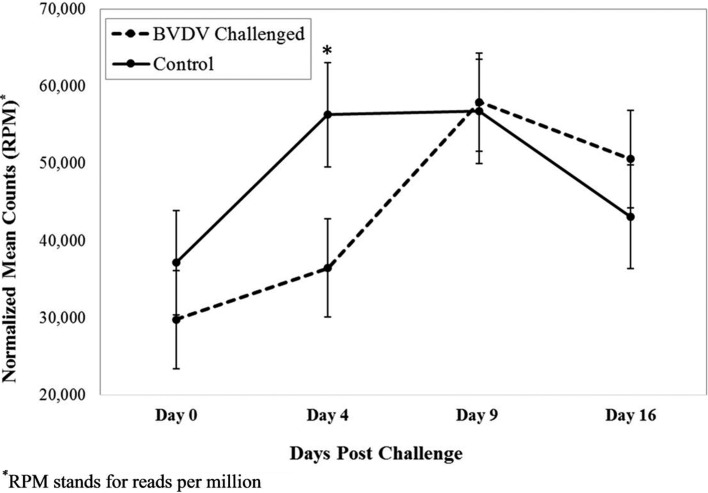
Interaction of time by treatment for 5’ tsRNA^GlyGCC^ (*p* = 0.04). Dashed line corresponds to cattle challenged with bovine viral diarrhea virus (BVDV). Solid line corresponds to control animals. Error bars represent standard error bars of the normalized means. An asterisk above a time point represents a significant (*p* = 0.01) difference in number of read counts between BVDV challenged and control animals at that time point.

The profile for 5’ tsRNA^GlyGCC^ by treatment group across time is shown in Figure 2. The count number was significantly higher in the control group than in the challenged group on day 4 (*p*=0.01). No significant difference was found between the treatment groups at any other time. While not reaching the level of significance, the count number in the control group tended to be higher than the challenged group on day 0. In the control group, the count number maintained a constant level from day 4 to 9. In contrast, the count number of the control group dipped on day 4 and rose to be similar to the control group on day 9.

## Discussion

Whole blood or serum samples are frequently collected for diagnostic purposes because sample collection, testing, and diagnosis may be performed while the animal remains alive. As research in this area expands, the role of extracellular tRNA-derived small RNAs, including tsRNAs in serum, and their association with gene regulation, disease, and viral infections continues to expand. This study identified more 5’ tsRNAs than 3’ tsRNAs (Cole *et al.*, 2009; Dhahbi *et al.*, 2013; Wang *et al.*, 2013; Casas *et al.*, 2015; Dhahbi, 2015;). Grouped by amino acid, the five most abundant 5’ tsRNAs (tsRNA^Gly^, tsRNA^Glu^, tsRNA^His^, tsRNA^Val^, and tsRNA^Lys^) in this study were also identified in sera from cattle and mice (Dhahbi *et al.*, 2013; Casas *et al.*, 2015).

The levels of two 5’ tsRNAs significantly differed between BVDV challenged and control calves 4 days post challenge. The down-regulation of 5’ tsRNA^GlyCCC^ and tsRNA^GlyGCC^ may represent a host immune response to the BVDV infection. These results continue to support the idea that tsRNAs act as immune signaling molecules (Dhahbi, 2015). Potentially, the down-regulation of these tsRNAs allowed for other sncRNAs, or solely regulated appropriate signals for an immune response to BVDV infection. By day 9, the BVDV challenged animals’ immune response returned to a more homeostatic state, as challenged animals show a lower viral load, increased lymphocyte counts, and increased number of reads for 5’ tsRNA^GlyCCC^ and tsRNA^GlyGCC^.

MicroRNAs were analyzed from the same dataset, and bta-miR-151-3p demonstrated similarities to 5’ tsRNA^GlyCCC^ and tsRNA^GlyGCC^ (Taxis *et al.*, 2017). At 4 days post challenge, 5’ tsRNA^GlyCCC^ and tsRNA^GlyGCC^ had lower read counts in challenged animals than controls, but read counts between treatment groups for bta-miR-151-3p were similar. Both the 5’ tsRNAs and bta-miR-151-3p increased in number of reads in challenged animals, with bta-miR-151-3p having a significantly higher read count in BVDV challenged animals than controls by 9 days post challenge. The comparison of results from the 5’ tsRNA^GlyCCC^, 5’ tsRNA^GlyGCC^, and bta-miR-151-3p may suggest that 5’ tsRNAs differ earlier during a BVDV challenge than microRNAs. A potential interaction between 5’ tsRNAs (5’ tsRNA^GlyCCC^ and tsRNA^GlyGCC^) and bta-miR-151-3p could be part of the signaling mechanism of the immune system against BVDV.

An alternative hypothesis to the down-regulation of the 5’ tsRNA^GlyCCC^ and tsRNA^GlyGCC^ may be a viral controlled response of the animal. tsRNAs are shown to inhibit translation initiation (Yamasaki *et al.*, 2009; Ivanov *et al.*, 2011; Yang and Schimmel, 2011). As the virus replicates in the animal it may initiate the down-regulation of 5’ tsRNA^GlyCCC^ and tsRNA^GlyGCC^. In turn, the genes that would typically be silenced by these tsRNAs, are expressed as normal. By day 9, as the host clears the virus, the tsRNAs return to a similar level as the non-infected animals.

Differences in 5’ tsRNA^GlyGCC^ were observed in previous studies. When studying respiratory syncytial virus in epithelial cells, 5’ tsRNA^GlyGCC^ was up-regulated in viral-infected cells (Wang *et al.*, 2013). In the present study, 5’ tsRNA^GlyGCC^ was down-regulated in serum of challenged animals. Respiratory syncytial virus is a member of the *Paramyxovirus* family, while BVDV is a member of the Flaviviridae family. Differences in viral replication strategies could be the reason why there are differences between the present study and results reported by Wang *et al.* (2013).

Further research is needed to support the profiles of both 5’ tsRNA^GlyCCC^ and tsRNA^GlyGCC^ in BVDV challenged animals. These experiments could include additional sample collections to better depict variations in the tsRNAs. As more *in vivo* challenge studies utilize animal samples, instead of cultured cells, and identify tsRNAs as well as other sncRNAs, the scientific community will gain knowledge of the function and importance of tsRNAs and sncRNAs to viral infections or immune response (Green *et al.*, 2016).

## Supplementary material

The following online material is available for this article:

Table S1 Total number of 3’ and 5’ tRNA-derived RNA halves with ≥ 1,000 or 5,000 total reads, respectively, sorted by tRNA, amino acid, and anticodon.

Table S2 5’ tRNA halves of tRNAProAGG and tRNAValAAC read, read length, and raw read counts among all animals.
