# Supplementary material for: Analysis of tRNA halves (tsRNAs) in serum from cattle challenged with bovine viral diarrhea virus
Source: Genet Mol Biol. 2019 Jun 27;42(2):374–9. doi: 10.1590/1678-4685-GMB-2018-0019 (PMC6726165; doi:10.1590/1678-4685-GMB-2018-0019)
Supplement: Supplementary file 1 [file 1415-4757-GMB-1678-4685-GMB-2018-0019-20190509-suppl1.pdf]

Supplementary Material to “Analysis of tRNA halves (tsRNAs) in serum from cattle challenged with bovine viral diarrhea virus”

Table S1 - Total number of 3’ and 5’ tRNA-derived RNA halves with ≥ 1,000 or 5,000 total reads, respectively, sorted by tRNA, amino acid, and anticodon.

tRNA	Anticodon	Number of reads
3’ tRNA-Asp	GTC	2,956
3’ tRNA-Val	TAC	2,529
3’ tRNA-Glu	TTC	1,564
3’ tRNA-Asn	GTT	1,246
5’ tRNA-Gly	CCC	44,021,842
5’ tRNA-Glu	TTC	20,826,124
5’ tRNA-His	GTG	9,758,491
5’ tRNA-Gly	GCC	9,356,830
5’ tRNA-Val	CAC	1,770,088
5’ tRNA-Val	TAC	782,195
5’ tRNA-Glu	CTC	599,433
5’ tRNA-Lys	CTT	237,120
5’ tRNA-Ala	CGC	110,904
5’ tRNA-Pro	AGG	90,567
5’ tRNA-Gln	TTG	60,023
5’ tRNA-Gln	CTG	58,384
5’ tRNA-Ala	AGC	52,911
5’ tRNA-Lys	TTT	44,323
5’ tRNA-Gly	TCC	33,024
5’ tRNA-Pro	TGG	14,590
5’ tRNA-Val	AAC	11,295
5’ tRNA-Cys	GCA	5,886
