# Supplementary material for: Analysis of tRNA halves (tsRNAs) in serum from cattle challenged with bovine viral diarrhea virus
Source: Genet Mol Biol. 2019 Jun 27;42(2):374–9. doi: 10.1590/1678-4685-GMB-2018-0019 (PMC6726165; doi:10.1590/1678-4685-GMB-2018-0019)
Supplement: Supplementary file 2 [file 1415-4757-GMB-1678-4685-GMB-2018-0019-20190509-suppl2.pdf]

Supplementary Material to "Analysis of tRNA halves (tsRNAs) in serum from cattle challenged with bovine viral diarrhea virus"

Table S2 - 5' tRNA halves of tRNAProAGG and tRNAValAAC read, read length, and raw read counts among all animals.

5' tRNA half	Read	Read Length	Raw Read Count*
tsRNA^{ProAGG}	GGCTCGTTGGTCTAGGGGTATGATTCTCGCT	31	35.206
	GGCTCGTTGGTCTAGGGGTATGATTCTCGCTT	32	27.216
	GGCTCGTTGGTCTAGGGGTATGATTCTCGC	30	14.871
	GGCTCGTTGGTCTAGGGGTATGATTCTCG	29	7.128
	GGCTCGTTGGTCTAGGGGTATGATTCTC	28	3.923
	GGCTTGTTGGTCTAGGGGTATGATTCTCGCT	31	887
	GGCTTGTTGGTCTAGGGGTATGATTCTCGCTT	32	664
	GGCTTGTTGGTCTAGGGGTATGATTCTCGC	30	382
	GGCTTGTTGGTCTAGGGGTATGATTCTCG	29	170
	GGCTTGTTGGTCTAGGGGTATGATTCTC	28	89
	GGCTCGTTGGTCTAGGGGTATGATTCTCGCTTA	33	30
	GGCTTGTTGGTCTAGGGGTATGATTCTCGCTTA	33	1
tsRNA^{ValAAC}	ATTTCCGTAGTGTAGTGGTTATCACGTTCGCC	32	3.157
	ATTTCCGTAGTGTAGTGGTTATCACGTTCGC	31	3.119
	GTTTCCGTGGTGTAGTGGTTATCACGTTCGC	31	1.422

	GTTTCCGTGGTGTAGTGGTTATCACGTTCGCC	32	1.335
	ATTTCCGTAGTGTAGTGGTTATCACGTTCGCCT	33	721
	GTTTCCGTGGTGTAGTGGTTATCACGTTCGCCT	33	304
	ATTTCCGTAGTGTAGTGGTTATCACGTTC	29	212
	GTTTCCGTAGTGTAGTGGTCATCACGCTCGC	31	184
	GTTTCCGTAGTGTAGTGGTCATCACGCTCGCCT	33	135
	GTTTCCGTAGTGTAGGGGTTATCACGTTCGCC	32	123
	GTTTCCGTAGTGTAGTGGTCATCACGCTCGCC	32	113
	GTTTCCGTAGTGTAGGGGTTATCACGTTCGC	31	112
	GTTTCCGTGGTGTAGTGGTTATCACGTTC	29	95
	ATTTCCGTAGTGTAGTGGTTATCACGTTCG	30	89
	GTTTCCGTAGTGTAGTGGTTATCACGTTCGCCTA	34	75
	GTTTCCGTGGTGTAGTGGTTATCACGTTCG	30	51
	GTTTCCGTAGTGTAGGGGTTATCACGTTCGCCT	33	29
	GTTTCCGTAGTGTAGGGGTTATCACGTTC	29	7
	GTTTCCGTAGTGTAGTGGTCATCACGCTC	29	3
	GTTTCCGTAGTGTAGTGGTTATCACGTTCGCCTAA	35	2
	GTTTCCGTAGTGTAGGGGTTATCACGTTCG	30	2
	GTTTCCGTAGTGTAGTGGTTATCACGTTCGCCTAACA	37	2
	GTTTCCGTGGTGTAGTGGTTATCACGTT	28	1
	GTTTCCGTAGTGTAGTGGTCATCACGCTCG	30	1
	ATTTCCGTAGTGTAGTGGTTATCACGTT	28	1
tsRNA^{GlyCCC}	GCATTGGTGGTTCAGTGGTAGAATTCTCGC	30	24.147.515
	GCATTGGTGGTTCAGTGGTAGAATTCTCGCC	31	18.514.197

GCATTGGTGGTTCAGTGGTAGAATTCTC	28	889.119
GCATTGGTGGTTCAGTGGTAGAATTCTCG	29	282.043
GCATTGGTGGTTCAGTGGTAGAATTCTCGCCT	32	73.292
GCATTGGTGGTTCAGTGGTAGAATTCTTGC	30	22.441
GCATTGGTGGTTCAGTGGTAGAATTCTT	28	18.758
GCATTGGTGGTTCAGTGGTAGAATTCTTGCC	31	16.773
GCATTGGTGGTTCAGAGGTAGAATTCTCGC	30	14.690
GCATTGGTGGTTCAGAGGTAGAATTCTCGCC	31	12.535
GCATTGGTGGTTCAGTGGTAGAATTCTCTC	30	7.770
GCATTGGTGGTTCAGTGGTAGAATTCTCTCC	31	6.547
GCGCCGCTGGTGTAGTGGTATCATGCAAGA	30	6.200
GCGCCGCTGGTGTAGTGGTATCATGCAAGATT	32	2.476
GCGCCGCTGGTGTAGTGGTATCATGCAAG	29	2.368
GCATTGGTGGTTCAGTGGTAGAATTCTCT	29	862
GCATTGGTGGTTCAGAGGTAGAATTCTC	28	595
GCATTGGTGGTTCAGTGGTAGAATTCTCGCCTCC	34	576
GCATTGGTGGTTCAGTGGTAGAATTCTCGCCTCCCACGCGGGAGACCCGG	50	535
GCATTGGTGGTTCAGTGGTAGAATTCTCGCCTCCC	35	491
GCGCCGCTGGTGTAGTGGTATCATGCAAGATTCCCATTCTTGCGACCCGG	50	355
GCATTGGTGGTTCAGTGGTAGAATTCTCGCCTC	33	300
GCATTGGTGGTTCAGTGGTAGAATTCTTG	29	284
GCATTGGTGGTTCAGTGGTAGAATTCTCGCCTCCCA	36	277
GCATTGGTGGTTCAGAGGTAGAATTCTCG	29	159
GCGCCGCTGGTGTAGTGGTATCATGCAAGATTCCC	35	119

GCGCCGCTGGTGTAGTGGTATCATGCAAGATTCCCATTCT	40	92
GCATTGGTGGTTCAGTGGTAGAATTCTTGCCT	32	74
GCGCCGCTGGTGTAGTGGTATCATGCAAGATTCC	34	67
GCGCCGCTGGTGTAGTGGTATCATGCAA	28	66
GCATTGGTGGTTCAGAGGTAGAATTCTCGCCT	32	49
GCGCCGCTGGTGTAGTGGTATCATGCAAGAT	31	42
GCATTGGTGGTTCAGTGGTAGAATTCTCGCCTCCCACGCGGG	42	38
GCGCCGCTGGTGTAGTGGTATCATGCAAGATTC	33	32
GCATTGGTGGTTCAGTGGTAGAATTCTCTCCT	32	22
GCGCCGCTGGTGTAGTGGTATCATGCAAGATTCCCATTCTTGC	43	15
GCGCCGCTGGTGTAGTGGTATCATGCAAGATTCCCATTC	39	15
GCGCCGCTGGTGTAGTGGTATCATGCAAGATTCCCA	36	13
GCATTGGTGGTTCAGTGGTAGAATTCTCGCCTCCCACG	38	9
GCGCCGCTGGTGTAGTGGTATCATGCAAGATTCCCATTCTTG	42	6
GCATTGGTGGTTCAGTGGTAGAATTCTCGCCTCCCACGCG	40	4
GCATTGGTGGTTCAGTGGTAGAATTCTCGCCTCCCACGTGGGAGACCCGG	50	3
GCATTGGTGGTTCAGTGGTAGAATTCTCGCCTCCCACGCGGGA	43	3
GCATTGGTGGTTCAGTGGTAGAATTCTCGCCTCCCACGCGGGAGA	45	3
TCCCTGGTGGTCTAGTGGCTAGGACTCTGC	30	2
GCATTGGTGGTTCAGTGGTAGAATTCTTGCCTCCC	35	2
GCGCCGCTGGTGTAGTGGTATCATGCAAGATTCCCATTCTTGCG	44	2
TCCCTGGTGGTCCAGTGGTTAAGATTCTGCG	31	1
TCCCTGGTGGTCCAGTGGTTAAGATTCTGCGC	32	1
GCATTGGTGGTTCAGTGGTAGAATTCTCTCCTCCCA	36	1

	GCGCCGCTGGTGTAGTGGTATCATGCAAGATTCCCATT	38	1
	GCATTGGTGGTTCAGTGGTAGAATTCTCGCCTCCCAC	37	1
	GCATTGGTGGTTCAGTGGTAGAATTCTCGCCTCCCACGCGGGAGAC	46	1
tsRNA^{GlyGCC}	GCATGGGTGGTTCAGTGGTAGAATTCTCGC	30	4.881.365
	GCATGGGTGGTTCAGTGGTAGAATTCTCGCC	31	4.153.691
	GCATGGGTGGTTCAGTGGTAGAATTCTC	28	186.874
	GCATGGGTGGTTCAGTGGTAGAATTCTCG	29	59.686
	GCATGGGTGGTTCAGTGGTAGAATTCTCGCCT	32	25.443
	GCATTGGTGGTTCAGTGGTAGAATTCTCGCCTG	33	18.629
	GCATTGGTGGTTCAGTGGTAGAATTCTCGCCTGCCA	36	10.330
	GCATTGGTGGTTCAGTGGTAGAATTCTCGCCTGCCACGCGGGAGGCCCGG	50	8.097
	GCATTGGTGGTTCAGTGGTAGAATTCTCGCCTGCC	35	7.368
	GCATTGGTGGTTCAGTGGTAGAATTCTCGCCTGC	34	1.424
	GCATGGGTGGTTCAGTGGTAGAATTCTCGCCTG	33	882
	GCATTGGTGGTTCAGTGGTAGAATTCTCGCCTGCCACGCG	40	645
	GCATTGGTGGTTCAGTGGTAGAATTCTCGCCTGCCACGCGGGAGG	45	276
	GCATTGGTGGTTCAGTGGTAGAATTCTCGCCTGCCACG	38	257
	GCATAGGTGGTTCAGTGGTAGAATTCTTGC	30	239
	GCATTGGTGGTTCAGTGGTAGAATTCTCGCCTGCCAC	37	224
	GCATTGGTGGTTCAGTGGTAGAATTCTCGCCTGCCACGCGGGAGGC	46	210
	GCATAGGTGGTTCAGTGGTAGAATTCTT	28	171
	GCATGGGTGGTTCAGTGGTAGAATTCTCGCCTGCCACGCG	40	155
	GCATTGGTGGTTCAGTGGTAGAATTCTCGCCTGCCACGCGGG	42	121
	GCATGGGTGGTTCAGTGGTAGAATTCTCGCCTGCCACGCGGGAGGCCCGG	50	83

GCATGGGTGGTTCAGTGGTAGAATTCTCGCCTGCC	35	82
GCATTGGTGGTTCAGTGGTAGAATTCTCGCCTGCCACGCGGGA	43	77
GCATTGGTGGTTCAGTGGTAGAATTCTCGCCTGCCACGC	39	63
GCATGGGTGGTTCAGTGGTAGAATTCTCGCCTGCCACG	38	59
GCATGGGTGGTTCAGTGGTAGAATTCTCGCCTGCCAC	37	48
GCATTGGTGGTTCAGTGGTAGAATTCTCACCTG	33	43
GCATGGGTGGTTCAGTGGTAGAATTCTCGCCTGCCA	36	36
GCATTGGTGGTTCAGTGGTAGAATTCTCGCCTGCCACGCGG	41	26
GCATGGGTGGTTCAGTGGTAGAATTCTCGCCTGCCACGCGGGAGGC	46	25
GCATGGGTGGTTCAGTGGTAGAATTCTCGCCTGCCACGCGGGAGG	45	24
GCATAGGTGGTTCAGTGGTAGAATTCTTGCT	31	23
GCATGGGTGGTTCAGTGGTAGAATTCTCGCCTGCCACGCGGG	42	22
GCATTGGTGGTTCAGTGGTAGAATTCTCACCTGCCA	36	21
GCATGGGTGGTTCAGTGGTAGAATTCTCGCCTGCCACGCGGGA	43	19
GCATTGGTGGTTCAGTGGTAGAATTCTTGCCTG	33	18
GCATGGGTGGTTCAGTGGTAGAATTCTCGCCTGC	34	16
GCATTGGTGGTTCAGTGGTAGAATTCTCACCTGCC	35	12
GCATTGGTGGTTCAGTGGTAGAATTCTCGCCTGCCACGCGGGAG	44	12
GCATTGGTGGTTCAGTGGTAGAATTCTTGCCTGCC	35	11
GCATTGGTGGTTCAGTGGTAGAATTCTTGCCTGCCA	36	8
GCATGGGTGGTTCAGTGGTAGAATTCTCGCCTGCCACGC	39	5
GCATAGGTGGTTCAGTGGTAGAATTCTTG	29	3
GCATGGGTGGTTCAGTGGTAGAATTCTCGCCTGCCACGCGG	41	2
GCATTGGTGGTTCAGTGGTAGAATTCTCACCTGC	34	2

GCATTGGTGGTTCAGTGGTAGAATTCTCACCTGCCAC	37	1
GCATGGGTGGTTCAGTGGTAGAATTCTCGCCTGCCACGCGGGAG	44	1
GCATTGGTGGTTCAGTGGTAGAATTCTTGCCTGC	34	1

*Raw read count includes the total number of reads represented among all animals, regardless of treatment group or days post challenge.
